# Recovery from chronic fatigue syndrome: a systematic review—heterogeneity of definition limits study comparison

**DOI:** 10.1136/archdischild-2020-320196

**Published:** 2021-04-12

**Authors:** Yasmin Moore, Teona Serafimova, Nina Anderson, Hayley King, Alison Richards, Amberly Brigden, Parisa Sinai, Julian Higgins, Caitlin Ascough, Philippa Clery, Esther M Crawley

**Affiliations:** 1 Centre for Academic Child Health, University of Bristol Faculty of Health Sciences, Bristol, UK; 2 Pennine Care NHS Foundation Trust, Ashton-under-Lyne, Lancashire, UK; 3 Collaboration for Leadership in Applied Health Research and Care West, National Institute for Health Research, Bristol, UK; 4 Royal United Hospital Bath NHS Trust, Bath, Bath and North East Somerset, UK

**Keywords:** adolescent health, occupational therapy

## Abstract

**Background:**

Paediatric chronic fatigue syndrome or myalgic encephalomyelitis (CFS/ME) is a common illness with a major impact on quality of life. Recovery is poorly understood. Our aim was to describe definitions of recovery in paediatric CFS/ME, the rate of recovery and the time to recovery.

**Methods:**

This systematic review included a detailed search of MEDLINE, EMBASE, PsycInfo and Cochrane Library between 1994 and July 2018. Inclusion criteria were (1) clinical trials and observational studies, (2) participants aged <19 years with CFS/ME, (3) conducted in Western Healthcare systems and (4) studies including a measure of recovery and time taken to recover.

**Results:**

Twelve papers (10 studies) were identified, involving 826 patients (range 23–135). Recovery rates were highly varied, ranging between 4.5% and 83%.

Eleven distinct definitions of recovery were used; six were composite outcomes while five used unidimensional outcomes. Outcome measures used to define recovery were highly heterogeneous. School attendance (n=8), fatigue (n=6) and physical functioning (n=4) were the most common outcomes included in definition of recovery. Only five definitions included a personal measure of recovery.

**Implications:**

Definitions of recovery are highly variable, likely secondary to differences in study design, outcomes used, follow-up and study populations. Heterogeneous definitions of recovery limit meaningful comparison between studies, highlighting the need for a consensus definition going forward. Recovery is probably best defined from the child’s own perspective with a single self-reported measure. If composite measures are used for research, there should be agreement on the core outcome set used.

## Abstract (precis)

Significant differences in definitions of recovery, result in highly varied reported recovery rates for chronic fatigue syndrome. Consensus definitions of recovery are needed, the authors recommend that recovery may be best defined from the child's own perspective.

**What is already known on this topic?**

- Chronic fatigue syndrome/myalgic encephalomyelitis (CFS/ME) is a complex, common illness which substantially impacts on quality of life. Recovery is poorly understood and defined.

**What this study adds?**

- Definitions of recovery are heterogeneous and recovery rates are variable. A consensus definition of recovery, focusing on patient perspective, is necessary to facilitate further research.

## Introduction

Paediatric chronic fatigue syndrome or myalgic encephalomyelitis (CFS/ME) is common (prevalence 0.1%–2.4%^1–4^) and has a substantial impact on children. Over 50% of children are bed-bound at some stage of their illness^4^ and lose a mean total of 1 year of school.^3 5^

Recovery from illness remains poorly defined and highly variable between different conditions. Much of the research examining recovery has been focused on adults^6^ and has demonstrated a range of highly personal targets that patients associate with recovery. In the context of mental health, it is increasingly acknowledged that a single end-point of ‘recovery’ is neither realistic nor relevant for many patients.^7^ A systematic review examining recovery in adult CFS/ME found a broad range of outcomes were used to quantify recovery, with the most commonly used measure being a brief global rating.^8^ Few of the included papers in this review considered the patients’ individual perception of what they considered ‘recovery’ to be.

Little is known about recovery in paediatric CFS/ME. Recovery is highly personal and is described by children with CFS/ME in many different ways.^9^ This is further complicated by lack of consensus on the definition of recovery. A 1997 systematic review of prognosis of CF and CFS populations, based on four small observational studies (n=15–31) using different definitions of recovery, reported that 54%–94% made a good or complete recovery at 13–72 months.^10^

The objective of our systematic review was to describe definitions of recovery in paediatric CFS/ME, within interventional trials and observational studies. We also aimed to describe what proportion recover, time to recovery and whether recovery rate differs between younger (<12 years) and older children.

## Methods

### Eligibility criteria

We included prospective studies (observational studies and clinical trials) which provided a measure of recovery (complete or partial) and time to recovery in children (<19 years) with CFS/ME, based within Western healthcare systems. We included studies where CFS/ME was defined using the CDC,^11^ NICE^12^ or Oxford criteria.^13^ Exclusion criteria included studies investigating children with fatigue due to other causes and chronic fatigue not defined using the aforementioned criteria. A pre-specified protocol was used and registered prospectively on PROSPERO (registration number: CRD42014009303).

### Search strategy

We conducted a systematic literature search of MEDLINE, EMBASE, PsychInfo and the Cochrane Library from 1994 to July 2014. We then updated this search to July 2018. We searched trial registration sites, hand searched reference lists and contacted authors for unpublished results. There were no language restrictions. Search terms for Ovid MEDLINE are presented in online supplemental appendix 1.

10.1136/archdischild-2020-320196.supp1 Supplementary data

### Study selection

Two researchers (EMC and AR) independently assessed titles and abstracts identified from electronic database searches from 1994 to 2014. The updated search from 2014 to 2018 was completed by two different researchers (PS and AB). All potentially relevant papers underwent independent full-text review by two different reviewers (YM and NA). Eligibility was assessed using predefined inclusion criteria. Data were independently extracted by two researchers (YM and NA) onto purpose-designed forms. Disagreements were discussed and resolved through discussion with a third researcher. Data were extracted on CFS/ME diagnostic criteria used, treatment/interventions provided, definition of recovery, study setting, recruitment method, date of the study and child characteristics. The rate of recovery was calculated as the number recovered divided by the number randomised for randomised studies or the number recovered divided by the number followed up for observational studies.

### Defining outcome measures

Outcomes were interpreted in the following way. Where single outcomes were measured (even if several were used in one study) and used to define recovery, this was termed a ‘unidimensional outcome’. Where numerical values for several unidimensional outcomes were combined, this was termed a ‘composite’ outcome. Where possible, the ‘proportion recovered’ has been reported for each dimension within a composite outcome.

### Data synthesis

Given the broad scope of this review, substantial heterogeneity was identified among selected studies, in terms of design, populations, intervention and outcome. Formal meta-analysis was not possible due to insufficient comparable data and so narrative synthesis was chosen as the most appropriate method.

## Results

### Description of studies

**Figure 1:**
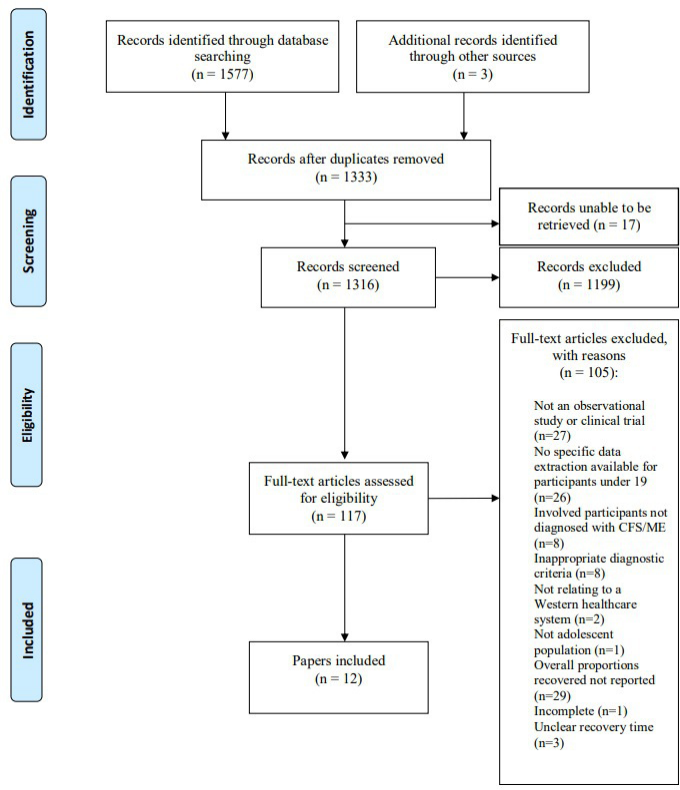
PRISMA flowchart.

Figure 1 describes the cumulative summary of search results from original and updated searches. Of 1577 papers identified, 117 underwent full-text review for eligibility. The final sample included 12 papers presenting data from 10 studies. For our description of definitions of recovery, each paper was the unit of interest, and for our description of recovery rates, each study was the unit of interest.

Table 1 describes the studies and papers included. Sample sizes varied between 23^14^ and 135.^15^ Mean age was between 14.2^16^ and 16 years^17^ (range 10–19 years). Three papers did not report details of participants’ ages.^14 18 19^ The majority of participants were female (range 63%^16^–90%^18 20^).

**Table 1:** Study characteristics

Author, year (reference)	Diagnostic criteria	Setting	Study design	Intervention	Sample at baseline (n)	Sample at follow-up n (% baseline)	Mean age (SD) (range)	Sex n/total at baseline (% female)	Follow-up (SD)(range)
Chalder *et al*, 2002^14^	CDC/Oxford	Tertiary	Observational	Family-focused CBT: up to 15 1-hour sessions	23	18 (78%)	NR (11–18)	20/23 (87%)	6 months
Chalder *et al*, 2010^23^	CDC/Oxford	Tertiary	RCT	Family-focused CBT (13 fortnightly 1-hour sessions) vs pyschoeducation	63	NR NR 59 (94%)	NR (13.0–17.0)	43/63 (68%)	3 months 6 months 12 months
Lloyd *et al*, 2012 (follow-up of Chalder *et al*, 2010)^21^	CDC/Oxford	Tertiary	RCT	Family-focused CBT (13 fortnightly 1-hour sessions) vs psychoeducation	63	44 (70%)	15 (1.7) (11–18)	32/63 (51%)	24 months
Crawley *et al*, 2011^3^	NICE	School	Observational	Specialist medical care offered to those diagnosed with CFS/ME	23	NR 19 (83%)	14.6 (1.5)	17/23 (74%)	1.5 months 6 months
Katz *et al*, 2009^18^	CDC	Secondary/tertiary	Observational	No intervention	39	36 (92%) 33 (85%)	NR	35/39 (90%)	6 months 18 months
Lim and Lubitz*l*, 2002^24^	CDC	Tertiary	Observational	Four-week intensive multidisciplinary inpatient programme	57	42 (74%)	15 (10–19)	50/57 (88%)	3.5 years (3 months to 5 years)
Nijhof *et al*, 2012^15^	CDC	Tertiary	RCT	Internet-based CBT vs usual care	135	131 (97%) 127 (94%)	15.9 (1.3)	111/135 (82%)	6 months 12 months
Nijhof *et al*, 2013 (follow-up of Nijhof *et al*, 2012)^22^	CDC	Tertiary	Long-term follow-up of RCT	Internet-based CBT vs usual care (with post-RCT crossover)	127	112 (88%)	15.8 (1.4)	105/127 (83%)	2.7 years (0.5)(1.7–3.8 years)
Rowe, 1999^19^	CDC	Tertiary	RCT	Gammaglobulin vs placebo	89	86 (97%)	NR	NR (NR)	56 months (25 months)(15–112 months)
Stulemeijer *et al*, 2005^20^	CDC	Tertiary	RCT	CBT (10 sessions) vs waiting list	69	62 (90%)	15.7 (1.3)	62/69 (90%)	5 months
Van Geelen *et al*, 2010^17^	CDC	Tertiary	Observational	Regular care	60	54 (90%)	16.0 (1.5)	NR (79.6%)	2.2 years (1.6)
Viner *et al*, 2004^16^	Modified CDC	Tertiary	Observational	Rehabilitative programme vs supportive care	78	56 (72%) 32 (41%) 24 (31%) 17 (22%)	14.2 (9–17)	49/78 (63%)	6 months 12 months 18 months 24 months

CBT, cognitive-behavioural therapy; NR, not reported; RCT, randomised controlled trial.

There were four randomised treatment trials. Two trials were also reported in long-term follow-up papers^21 22^—therefore six papers in total. One of these two follow-up papers did not report separate recovery rates for intervention and control groups.^22^ Children were offered cognitive-behavioural therapy (CBT: individual only, family-focused, telephone or internet based),^15 20–23^ psychoeducation^21 23^ and gammaglobulin therapy.^19^ There were six observational studies. One involved an inpatient treatment programme,^24^ the remainder involved outpatient treatments. Outpatient treatments included CBT (family-focused^14^), specialist medical care,^3^ graded activity and exercise programmes with sessions targeting ‘practical management issues’^16^ and ‘regular care’.^17^ One observational study did not involve any intervention.^18^ One study identified children after school-based screening.^3^ Mean follow-up for included studies was wide (range 1.5 months^3^ to 56 months^19^).

### Definition of recovery

Recovery was explicitly defined in four papers.^15 17 21 22^ In the remaining papers, recovery was implied from the outcome measures reported. In studies using patient-reported outcomes, those who were ‘completely back to normal’ or whose symptoms ‘resolved’ were assumed to be ‘recovered’.^24^

Definitions of recovery used were varied (table 2). Twelve papers used 11 different definitions. Of these, three used one outcome to define recovery^3 19 23^ and nine reported several outcomes.^14–18 20–22 24^ In seven papers, outcomes were combined into a composite measure of recovery.^14–18 21 22^ Three of these seven had a composite outcome as their primary outcome.^14 17 18^ The remaining four had unidimensional primary outcomes.^15 16 21 22^ Three reported both composite outcomes and separate recovery rates for each domain within the composite outcome.^15 17 22^

**Table 2:** Study results

Author, year (reference)	Domain	Definition of recovery/partial recovery	Detailed notes on outcome	Proportion recovered
Unidimensional outcome				
Chalder *et al*, 2010^23^	School attendance	School attendance ≥70%	Attendance at school/work over a 2-week period as a percentage of expected attendance at 6-month follow-up. Information obtained from parents. Dichotomised outcome set at 70% as many healthy adolescents are not attending full-time school/college at this age	**Family-focused CBT vs psychoeducation**: 56% (18/32) vs 29% (9/31) at 3 months 66% (21/32) vs 58% (18/31) at 6 months 59% (19/32) vs 65% (20/31) at 12 months
Crawley *et al*, 2011^3^	School attendance	Full recovery: full-time attendance	Ascertained at assessment using single-item inventory and through follow-up questionnaires. full time not quantified	32% (6/19) at 1.5 months 63% (12/19) at 6 months
Rowe, 1999^19^	Self-rated improvement	‘Participants considered they were well’	Questionnaire	60% at mean 56 months (range 15–112 months)
Lim and Lubitz, 2002^24^	Physical activity	‘Completely back to normal’ assumed to indicate recovery	Self-administered questionnaire with multiple choice answers	4/41 (10%) immediately following intervention 8/42 (19%) at long-term follow-up (3–5 years)
	Fatigue	‘Resolved’ assumed to indicate recovery		6/41 (15%) following intervention
	Memory and concentration	‘Better or resolved’ assumed to indicate partial recovery or recovery		38/42 (90%) during admission
	Social activities	‘Back to normal or mildly limited in social activities’ assumed to indicate recovery or partial recovery		22/40 (52%) following intervention 36/41 (88%) at long-term follow-up (3–5 years)
	School attendance	‘Full time’ assumed to indicate full recovery		6/39 (15%) following intervention 20/41 (49%) at long term follow-up
Stulemeijer *et al*, 2005^20^				CBT vs waiting list
	Fatigue	Reliable change index >1.96 and fatigue score <35.7 on fatigue subscale of CIS-20*	All measures self-reported	21/35 (60%) vs 7/34 (21%) at 5 months
	Physical functioning	Increase of ≥50 or an end score of ≥75 on physical functioning subscale of SF-36†		22/35 (63%) vs 8/34 (24%) at 5 months
	School attendance	Full-time school attendance		19/33 (58%) vs 10/34 (29%) at 5 months
	Self-rated improvement	‘Completely recovered’ or ‘feel much better’ on 4-point Likert scale		25/35 (71%) vs 15/34 (44%) at 5 months
Composite				
Chalder *et al*, 2002^14^	Fatigue school attendance	Improved: attendance at school at ≥75% of the time and a score of <4 on Chalder Fatigue Scale‡	No explanation of how attendance measured	83% (15/18) at 6 months
Katz *et al*, 2009^18^	Clinical evaluation (physical examination, history, blood tests)	Recovered based on complete history, physical examination and laboratory screening	Assessment of recovery status through telephone screening interview	31% (11/36) at 6 months 52% (17/33) at 18 months
Lloyd *et al*, 2012 (follow-up of Chalder *et al*, 2010)^21^	Fatigue school attendance	Recovery: score ≤18 on the Chalder Fatigue Scale‡ questionnaire and school attendance ≥70%	Score ≤18 chosen in line with cut-off for fatigue recovery defined by^25^ school attendance assessed by self-reported hours as percentage of expected attendance per week. Cut-off ≥70% used as at this age many healthy adolescents are not attending school or college full time	**Family-focused CBT vs psychoeducation:** 79% vs 64% at 24 months
Nijhof *et al*, 2012^15^				CBT vs usual care
	Fatigue	Fatigue severity score <40 on CIS-20*	Recovery was defined post hoc, in relation to healthy peers±2 SD	57/67 (85%) vs 17/64 (27%) at 6 months
	School attendance	School absence of ≤10% in past 2 weeks	Attendance measured as proportion of classes attended, expressed as a percentage of normal school schedule	50/67 (75%) vs 10/64 (16%) at 6 months
	Physical functioning	Physical functioning score ≥85% on CHQ-CF87 subscale§		52/67 (78%) vs 13/64 (20%) at 6 months
	Self-rated improvement	Self-rated as completely recovered or feeling much better¶		42/57 (63%) vs 5 (8%) at 6 months
	Composite			63% (42/67) vs 8% (5/64) at 6 months 64% (41/64) vs 8% (5/63) at 12 months
Nijhof *et al*, 2013 (follow-up of Nijhof *et al*, 2012)^22^	Fatigue School attendance Self-rated improvement Physical functioning	Recovery: fatigue severity score <40 on CIS-20†, physical functioning score ≥85% on CHQ-CF87 subscale§, school absence ≤10% in past 2 weeks and self-rated as completely recovered or feeling much better¶	Recovery was defined post hoc, in relation to healthy peers±2 SD. School attendance measured as the proportion of classes attended, expressed as a percentage of the normal school schedule	55% (62/112) at 12 months** 59% (66/112) at mean of 2.7 years (range 1.7–3.8 years)
Viner *et al*, 2004^16^	Global wellness School attendance	Resolution: wellness†† score of ≥90†† and school attendance ≥95%	Average school attendance in previous 3 months measured by families with data verified by telephone contact to school/college or percentage of required weekly contact calculated for those in college	**Programme vs supportive care** 43% (11/26) vs 4.5% (1/22) at 6–24 months
Van Geelen *et al*, 2010^17^	Fatigue	Score <40 on fatigue subscale of CIS-20*	Cut-off score of 40 chosen as it is the mean plus 2 SD of subjective fatigue distribution in healthy adolescents	30/54 (55.6%) at mean 2.2 years
	Physical functioning	Score >65 on CHQ-CF87 physical functioning subscale§		38/54 (70.4%) at mean 2.2 years
	Composite			52% (28/54) at mean 2.2 years

*Checklist Individual Strength-20 (CIS-20) consists of 8 items on a 7-point scale with scores ranging from 8 (no fatigue) to 56 (severely fatigued).

†The SF-36 has scores ranging between 0 (maximal physical limitation) and 100 (ability to do vigorous activity), with a subscale for measuring physical functioning.

‡Chalder Fatigue Scale: an 11-item questionnaire measuring physical and mental fatigue on a 4-option Likert continuum to give a total fatigue score. Scoring is bimodal (range from 0 to 11). A score of >4 indicates ‘severe fatigue’.

§CHQ-CF87 is an 87-item questionnaire with a score range of 0%–100%, with 0 indicating maximal physical limitation while 100% indicates the ability to do all activities.

¶Self-rated Improvement was measuring by using a 4-item tool in which patients can indicate whether they have “completely recovered”, “feel much better”, “have the same complaints” or “have become much worse” compared with the measurement before commencement of CFS treatment.

**Paper did not report separate recovery rates for intervention and control arms at long term follow-up.

††Self-rated single item global health wellness score provides a subjective assessment of overall physical and mental well-being and is considered a measure of overall health and quality of life.

Of the nine papers using several unidimensional definitions, seven included change in fatigue,^14 15 17 20–22 24^ seven incorporated school attendance^14–16 20–22 24^ and five used improvement in physical function.^15 17 20 22 24^ One used clinical examination and investigations to identify recovered participants.^18^ Another paper used self-reported ratings on physical activity, fatigue, cognition and social activities. Those who reported being ‘completely back to normal’ or whose symptoms ‘resolved’ were assumed to be ‘recovered’ and symptoms ‘better or resolved’ was assumed to indicate partial recovery or recovery.^24^ One paper included a measure of global wellness.^16^ Patient-reported measures of improvement or ‘recovery’ were used in six papers.

Of the three papers using only one unidimensional definition, two used school attendance^3 23^ and one used self-rated improvement on a questionnaire.^19^ In our analysis of the latter, we interpreted self-rated improvement where “participants considered they were well” as an indication that they had recovered.^19^

Substantial heterogeneity among outcome measures was noted. There were 11 different outcome measures used within the studies included (table 2). Different methods and thresholds were used to record each outcome. Physical functioning was measured by the Child Health Questionnaire—child form 87 (CHQ-CF87),^15 17 22^ or an unspecified “self-administered questionnaire with multiple choice answer” in one study.^24^ Fatigue was measured using the Chalder Fatigue Scale,^14 21^ fatigue severity sub-scale of CIS-20^15 17 22^ and the unspecified questionnaire.^24^ School attendance was measured by parents, telephone contact with the school, self-report and other unreported methods. Different thresholds were assumed to indicate recovery, from >70%^23^ to full-time school attendance.^3^ Cut-off scores used were variable. On the CIS-20, a score of <40 was required.^15 17 22^ On physical functioning subscale CHQ-CF87, scores of ≥85^15 22^ and >65^17^ were used.

Two of the 12 papers reported their rationale for their chosen definition of recovery.^16 21^ Lloyd *et al* 21 used a cut-off on the Chalder Fatigue Scale previously used in the PACE trial.^25^ School attendance was also incorporated due to the impact of CFS/ME on educational and social development. Viner *et al* 16 incorporated a Global Wellness score as a marker for overall health and quality of life, previously used in adult CFS/ME studies. School attendance was used as a surrogate marker of an individual’s “functional status” and ability to “participate in normal life”. The remaining 10 papers did not explain their choice of outcome(s) used to define recovery.

### Proportion of children who recover

Recovery rates at follow-up ranged from 4.5%^16^ to 83%^14^ (mean follow-up time 6–56 months) across all reported results from the 12 papers, including both treatment and control arms (table 2). Within the RCTs which reported separate recovery rates, children allocated to interventions had higher recovery rates compared with controls. Within intervention arms, recovery rates ranged from 43% to 79% (follow-up time 5–24 months).^15 20 21 23^ In control groups, recovery rates ranged from 4.5%^16^ to 64%^21^ (mean follow-up time 6–24 months). Recovery rates were lowest where the control arm constituted ‘supportive care’, ‘usual care’ or ‘waiting list’, ranging from 4.5%^16^ to 21%.^20^ In one RCT (reported in two papers), the control arm constituted psychoeducation. Here, recovery rates ranged from 29% to 65%^23^ (mean follow-up 3 months to 24 months). Within observational studies, recovery rates ranged from 19% to 83% (mean follow-up time 6 months to 3.5 years).

Recovery rates were lower when composite outcomes were used (table 2). Chalder showed that 95% of children were meeting recovery thresholds at 6-month follow-up when defined as school attendance >75%.^14^ However, this recovery proportion fell to 83% when school attendance was combined with fatigue score >4 on Chalder Fatigue Scale as part of a composite definition of recovery.^14^

### Do recovery rates change over time?

Exact time to recovery was not reported in any of the included papers.

Six papers specifically reported recovery at 6 months.^3 14–16 18 23^ Recovery rate ranged from 58% to 83% in the intervention groups.^14 15 23^ All interventions involved CBT and one involved psychoeducation.^23^ Recovery was lower in children receiving usual care or no intervention at 6 months (range: 8%^15^ to 31%^18^).

Two papers reported recovery at 12 months.^15 23^ A recovery rate of 64% was reported in those receiving CBT, compared with 8% in those receiving usual care.^15^ Recovery rates were stable between 6 and 12 months in both arms.^15^ The other found 59% recovered following CBT, while 65% recovered after psychoeducation.^23^

Six papers reported recovery rates at multiple time points.^3 15 18 22–24^ Four of these showed increased recovery rates over time.^3 18 22 24^ One showed improvement in the control arm (psychoeducation) but lower recovery rates in the intervention arm between 6 and 12 months.^23^

The included papers primarily involved teenage participants. Few included younger children (<12 years old); where they were included, data specifically for this population were not available. No papers specifically explored recovery in a primary school–aged population. As a result, we were unable to comment on whether recovery rates differ in younger populations.

## Discussion

This is the first contemporary systematic review exploring both recovery rates and definitions of recovery in paediatric CFS/ME in those receiving either specialist care or no intervention. A broad range of definitions of recovery were identified, which, in part, resulted in highly varied recovery rates. School attendance and fatigue represented the two most common measures used to define recovery. The majority of studies defined recovery according to multiple parameters. Few studies included in our review examined children’s perceptions of recovery or considered which aspects were most meaningful to them.

Across all studies, children receiving no treatment had recovery rates ranging from 21% to 52%. Children receiving ‘usual care’ had recovery rates ranging from 4.5% to 63%. Recovery rates were higher among children offered intensive interventional treatment, ranging from 43% to 83%. Highly varied recovery rates are likely, in part, secondary to the heterogeneous definitions of recovery used and the broad range of outcome measures used within these definitions. Interpretation is also challenging due to lack of clearly defined interventions/controls and varied follow-up time.

### Strengths and weaknesses

Strengths of this review include its comprehensive search strategy and rigorous study selection progress. A detailed search was conducted in four databases, identifying data published over the last 24 years. Reference lists and trial registration websites were hand searched to reduce publication bias. Strict eligibility criteria facilitated improved comparison of results. We were unable to locate 17 papers. We included papers in any language but excluded studies conducted outside Western healthcare systems. This limits interpretation of our results in other healthcare settings.

Interpretation was challenging due to the variety of definitions of recovery used, with different methods and thresholds used to measure and define outcomes. The clinical significance or rationale used to determine thresholds was rarely explained or justified.

Nine of the 10 studies recruited participants from tertiary care. One study used CFS/ME severity thresholds (CIS-20 score below cut-off) when recruiting participants, excluding participants with less severe symptoms.^17^ This limits the generalisability of our results as the populations included may over-represent those most severely affected by CFS/ME with a worse prognosis.^3^

Certain groups of children affected by CFS/ME were not evaluated in the included studies. Four studies excluded participants with comorbid psychiatric conditions.^14 15 20 23^ This may represent an attempt to avoid confounding due to improvement in comorbid mental health disorders, rather than CFS/ME recovery. This is particularly important given several studies involved psychological interventions. However, given the high prevalence of comorbid mental health disorders within the CFS/ME population,^26^ this may limit the applicability of these results. As studies included did not assess children under 10 years old, we cannot comment on recovery rates in this age group. This warrants further research going forward.

Substantial heterogeneity will exist among the quality, quantity and nature of standard treatment provided to those receiving ‘usual care’, due to differences in local provisions.^15 16 22^ Likewise, substantial heterogeneity between the intensity, length, mode of delivery and approach of interventions made comparison challenging. Recovery rates were lower when composite outcome measures were used to define recovery. With the exception of one study,^14^ recovery was higher in interventional trials compared with observational studies. Papers variably reported the number of recovered children as a proportion of either those randomised or those followed up. This skewed our ability to interpret recovery rates.

The data presented in the literature reviewed meant we were unable to comment on two of our initial review questions that discussed the extent to which participant age determined prognosis and the time to recovery.

### Results in the context of previous literature

This paper is the first and only contemporary systematic review of recovery in paediatric CFS/ME. Recovery rates reported here are consistent with the only other systematic review conducted over 20 years ago in 1997, which found 54%–94% of children reported they had recovered or made a ‘definite improvement’ over 13–72 months. The 1997 review exclusively used patient-reported or parent-reported outcomes. In contrast, our findings demonstrate the use of a range of different measures, including objective and patient-reported measures, to classify recovery. This review also solely focuses on paediatric CFS/ME unlike the previous review which included both adult and paediatric populations.

‘Recovery’ is a complex concept which varies between patients.^9^ There is currently no consensus definition for recovery in paediatric CFS/ME which limits comparison between studies. Of the papers included in our review, nine used multiple outcomes to measure recovery but only five included the patients’ own perspective to address the component of ‘personal recovery’.^15 16 19 20 22^ The majority of included studies used at least one objective measure of recovery, such as school attendance. Patient-reported measures are likely to be crucial in developing a definition of recovery which is meaningful to children and families.^9 27 28^ Data from this review also suggest that recovery rates may be overestimated if single outcome measures are used, consistent with adult CFS/ME studies,^29^ and also may highlight selective rather than global improvement. This suggests that in the absence of a personal measure of recovery, composite measures may more adequately encompass the complexity of ‘recovery’.

## Conclusions

Substantial heterogeneity in definitions of recovery, and outcome measures used therein, likely results in highly varied recovery rates across different studies and definitions. Although there is a suggestion of improved recovery rates among children offered specialist care, this review did not seek to examine intervention effectiveness. Going forward, there is a need to develop a consensus definition of ‘recovery’ in paediatric CFS/ME.

Given the personal and varied experiences of children with CFS/ME, what children feel define recovery is probably best defined from the child’s own perspective with a single self-reported measure of whether they feel they have recovered or not. This could also allow children to quantify their experience on a spectrum, between ‘unwell’ and ‘well’, which may be more easily intelligible compared with describing themselves as ‘recovered’. That said, a single outcome measure is vulnerable to distortion by encompassing multiple aspects of a child’s overall health status. Using multiple outcomes may help identify specific limitations or challenges children face secondary to their illness. Composite measures may, therefore, be more useful in a research context. If composite measures encompassing multiple parameters are used for research, these should be agreed as a core outcome set that is relevant to the child, their families and those around them.

## Data Availability

No additional data are available. All data discussed here has been published previously.
