# Supplementary material for: Recovery from chronic fatigue syndrome: a systematic review—heterogeneity of definition limits study comparison
Source: Arch Dis Child. 2021 Apr 12;106(11):1087–94. doi: 10.1136/archdischild-2020-320196 (PMC8543221; doi:10.1136/archdischild-2020-320196)
Supplement: Supplementary data [file archdischild-2020-320196supp001.pdf]

Appendix 1: Search strategy for Ovid MEDLINE

Search terms

1. Paediatric

(adolesc* OR preadolesc* OR pre-adolesc* OR boy* OR girl* OR child* OR infan* OR preschool* OR pre-school* OR juvenil* OR minor* OR school* OR pe?diatri* OR pubescen* OR prepubescen* OR prepubescen* OR puberty OR student* OR teen* OR young* OR youth* OR school* OR high-school OR highschool OR college OR undergrad* OR campus* OR classroom*) child* or infant* or minor* or sibling* or adolesc* or preadolesc* or pre-adolesc* or boy* or girl* or preschool* or pre-school* or juvenil* or school* or schoolchild* or school child* or p?ediatri* or pubescen* or prepubescen* or pre-pubescen* or puberty* or student* or teen* or young* or youth* or highschool* or high-school* or college* or undergrad* or campus* or classroom*) (adolescen$ or boy$ or child$ or children or early life or girl$ or infan$ or juvenile$ or lifecourse or life course or life-course or minor$ or preschool$ or pre-school$ or pre school$ or pediatr$ or paediatr$ or student$ or schoolchild$ or teenage$ or school child$ or teenage$ or young or youth$).tw. or exp Adolescent or exp Child or exp Child, Preschool or exp Infant or exp Life Change Events or exp Minors or exp Pediatrics or exp Students/

2. CFS/ME

Chronic Fatigue Syndrome.tw myalgic encephal*.tw. CFS.tw ME.tw chronic fatigue.mp. fatigue syndrome$.mp. exp Fatigue Syndrome, Chronic/ chronic fatigue.mp. myalgic encephalomyelitis.mp.

3. Recovery

Prognosis Outcome Trial Cohort Case-control Longitudinal
